# Electrochemical Enhancement of Photocatalytic Disinfection on Aligned TiO_2_ and Nitrogen Doped TiO_2_ Nanotubes

**DOI:** 10.3390/molecules22050704

**Published:** 2017-04-28

**Authors:** Cristina Pablos, Javier Marugán, Rafael van Grieken, Patrick Stuart Morris Dunlop, Jeremy William John Hamilton, Dionysios D. Dionysiou, John Anthony Byrne

**Affiliations:** 1 Department of Chemical and Environmental Technology, ESCET, Universidad Rey Juan Carlos, c/Tulipán s/n, 28933 Móstoles, Madrid, Spain; javier.marugan@urjc.es (J.M.); rafael.vangrieken@urjc.es (R.v.G.); 2 Nanotechnology and Integrated BioEngineering Centre (NIBEC), Ulster University, Newtownabbey BT37 0QB, Northern Ireland, UK; psm.dunlop@ulster.ac.uk (P.S.M.D.); jwj.hamilton@ulster.ac.uk (J.W.J.H.); j.byrne@ulster.ac.uk (J.A.B.); 3 Environmental Engineering and Science program, University of Cincinnati, 705 Engineering Research Center, Cincinnati, OH 45221, USA; dionysios.d.dionysiou@uc.edu

**Keywords:** titania nanotubes, nitrogen-doped nanotubes, photoelectrocatalysis, *E. coli*, visible light

## Abstract

TiO_2_ photocatalysis is considered as an alternative to conventional disinfection processes for the inactivation of waterborne microorganisms. The efficiency of photocatalysis is limited by charge carrier recombination rates. When the photocatalyst is immobilized on an electrically conducting support, one may assist charge separation by the application of an external electrical bias. The aim of this work was to study electrochemically assisted photocatalysis with nitrogen doped titania photoanodes under visible and UV-visible irradiation for the inactivation of *Escherichia coli*. Aligned TiO_2_ nanotubes were synthesized (TiO_2_-NT) by anodizing Ti foil. Nanoparticulate titania films were made on Ti foil by electrophoretic coating (P25 TiO_2_). N-doped titania nanotubes and N,F co-doped titania films were also prepared with the aim of extending the active spectrum into the visible. Electrochemically assisted photocatalysis gave higher disinfection efficiency in comparison to photocatalysis (electrode at open circuit) for all materials tested. It is proposed that electrostatic attraction of negatively charged bacteria to the positively biased photoanodes leads to the enhancement observed. The N-doped TiO_2_ nanotube electrode gave the most efficient electrochemically assisted photocatalytic inactivation of bacteria under UV-Vis irradiation but no inactivation of bacteria was observed under visible only irradiation. The visible light photocurrent was only a fraction (2%) of the UV response.

## 1. Introduction

Chlorination is an effective approach for the disinfection of water; however, it can lead to the formation of disinfection by-products e.g., trihalomethane, which can be mutagenic and carcinogenic. Furthermore, some species of pathogenic microorganisms are resistant to chlorination and ozonation [1]. As a consequence, new technologies have been developed to overcome the drawbacks of current disinfection processes. Heterogeneous photocatalysis is an advanced oxidation process (AOP) which can operate under ambient temperature and pressure, and oxygen from the air can be utilized as the oxidant without the addition of consumable chemicals. If one can use solar energy to drive the photocatalytic process then it becomes a truly clean technology. Since the early work of Matsunaga et al. [2], TiO_2_ photocatalysis has been reported by many research groups to be effective for the inactivation of a wide range of microorganisms in water [3,4].

The use of TiO_2_ suspensions for photocatalysis involves an additional post treatment step to separate the particles from the treated water, which may increase the complexity and cost of treatment. Another disadvantage of suspension systems is low quantum yield for hydroxyl radical generation due to the recombination of charge carriers. Electrochemically enhanced photocatalysis using TiO_2_ photoanodes is a potential solution to improve charge carrier separation and this approach utilizes immobilized photocatalyst without the need for post-treatment separation. The application of an external electrical potential to the TiO_2_ anode can improve charge carrier separation and thus reduce the rate of recombination. Under irradiation, photogenerated valence band holes migrate to the semiconductor surface where the water oxidation occurs, producing hydroxyl radicals (∙OH), and the photogenerated conduction band electrons migrate or diffuse to the supporting electrode, from where they are driven to the counter electrode and passed on to molecular oxygen creating superoxide radical anion (O_2_^·−^). Subsequent reduction reactions yield hydrogen peroxide (H_2_O_2_) and ∙OH. The use of electrochemically assisted photocatalysis for the disinfection of water and the degradation of organic pollutants has been previously reported [5,6,7]. One of the important advantages of the application of electrochemically assisted photocatalysis with respect to the inactivation of microorganisms is that mass transport may be enhanced through electromigration of negatively charged bacteria to a positively biased photoanode [8,9,10]. 

Nanoengineering of the titania (TiO_2_) electrodes is an interesting approach to improve the efficiency for electrochemically assisted photocatalytic disinfection. The interest in the potential use of titanium dioxide nanotubes as photoanodes has been increasing. They may exhibit better photoelectrolytic properties compared with nanoparticle films, due to the short diffusion path for photogenerated holes and a direct path for photogenerated electrons to the supporting electrodes [11,12,13]. In 1999, anodically grown self-organized TiO_2_ nanotubes were reported by Zwilling et al. [14]. These nanotubes can be prepared by anodic oxidation of a Ti substrate in fluoride containing electrolytes. TiO_2_ nanotubes remain attached on the substrate and therefore can be used directly as photoanodes. It has been reported that photoelectrolytic degradation of chemical contaminants in water is more effective with the TiO_2_-NT electrodes as compared to TiO_2_ nanoparticulate electrodes [12,15,16]. However, few studies have been conducted to date on the use of nanotubular TiO_2_/Ti for electrochemically assisted photocatalysis in water disinfection [9,17,18,19,20].

A major drawback of TiO_2_ for solar applications is that it has a wide band gap (anatase = 3.2 eV) which means that it absorbs in the UV domain and only 4% of solar photons are of sufficient energy to excite TiO_2_. Many efforts have been made to modify titania (particles, films, and nanotubes) in order to extend the action spectrum into the visible domain of the electromagnetic spectrum as around 45% of solar photons are in the visible wavelength range [13,21,22]. TiO_2_ doping with non-metals, in particular with nitrogen, has attracted great interest since Asahi and co-workers [23] reported in 2001 that N-doped TiO_2_ showed visible light photocatalytic activity. They reported that the mixing of N 2p with O 2p states in valence band results in the narrowing of the TiO_2_ band-gap and shifting absorption onset of TiO_2_ to lower energies. However, since heavy doping of the metal oxide semiconductor is required for narrowing the band gap [24,25], other mechanisms regarding the causes leading the absorption edge of TiO_2_ to be shifted towards the visible region have been proposed. Several authors [26,27,28] have suggested that sub-band gap excitation is due to isolated N 2p states located above the valence band maximum as a result of N-doping. Other groups have pointed out an increase in the absorption of visible light due to the presence of oxygen vacancies, induced as a consequence of N doping, which give rise to the formation of Ti^3+^ defect states, also called color centers [28,29]. There is lack of consensus since these states have been reported to be lying just below the conduction band [27,29,30,31,32] but also just above the valence band [25,32,33,34,35]. Although some authors such as Yang et al. [36] and Nakamura et al. [37] have reported that these color centers may act as electron trapping sites, leading to new photo excitation processes, they have traditionally been reported as responsible for hole trapping, leading to charge carrier recombination [26,28,33,38]. It has been reported that while Ti^3+^ color centers can give rise to visible light absorption, they may not contribute to visible photocatalytic activity [38]. Furthermore, co-doping with N and F has been reported to yield visible light activity. The presence of fluorine as a dopant induces formation of shallow Ti^3+^ donor levels a few tenths of electron volts below the conduction band [39]. Calculations suggest that the co-doping N-TiO_2_ with fluorine reduces the number of oxygen vacancies as compared to nitrogen doping alone [40].

It is worth noting that several reports have been published concerning the potential use of N-TiO_2_ photocatalysts for disinfection applications [41]. However, no papers concerning electrochemically assisted photocatalytic disinfection involving N-doped titania nanotubes have been found. Therefore, the aim of this work is to study the effect of the application of an electric potential bias on the photocatalytic inactivation efficiency of *E. coli* under simulated solar irradiation by using TiO_2_ particulate films, sol gel films, and N-doped titania nanotubes as photoanodes. In this work, N,F-TiO_2_ has been used as a reference for a reported visible light active photocatalyst material [42].

## 2. Results and Discussion

### 2.1. Electrode Characterization

**Figure 1:**
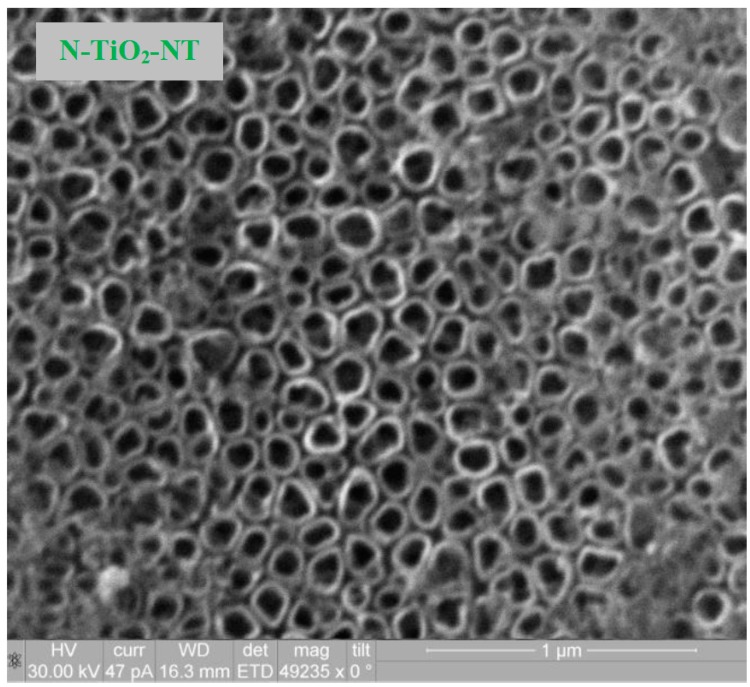
SEM image of titania nanotubes (N-TiO_2_-NT) grown by anodization of Ti foil, followed by annealing in ammonia atmosphere.

SEM analysis of TiO_2_ nanostructures obtained by anodization of Ti foil were carried out for both, undoped and doped titania nanotube samples. The SEM image for the N-doped titania NT electrode is shown in Figure 1 as an example. There was no discernible difference observed by SEM between the titania nanotubes annealed in air (TiO_2_-NT) and those annealed in NH_3_ (N-TiO_2_-NT). 

The nanotubes are observed as discrete, hollow, and cylindrical tube-like features (both undoped and N-doped). They are uniformly distributed throughout the sample and densely populated. It must be noted that the undoped and doped nanotubular layers resist heat treatment. They show a shape similar to stacked rings, which has already been observed in the case of tubes grown in aqueous electrolytes containing fluoride ions [43,44]. For both samples, not only the diameter of nanotubes (ca. 100 nm) but also the wall thickness (ca. 8 nm) was similar, which is in agreement with the dimensions reported by other researchers using anodizing potentials between +20 to +25 V [12,43,44,45,46]. Previous XRD analysis of TiO_2_-NTs showed that annealing at 450 °C in air gives the anatase crystal phase [47]. Annealing the NTs in NH_3_ at 450 °C does not alter the crystal phase. SEM analysis of the electrophoretically deposited P25 electrode (P25 TiO_2_) has already been reported [48]. A highly porous fractured appearance was obtained. The P25 TiO_2_ is an 80:20 ratio of anatase to rutile and this does not change following annealing at 450 °C. The N,F-TiO_2_ used in this work has been previously characterized by XRD and has been found to consist predominantly of the anatase phase [42]. The overall surface morphology of the N,F-TiO_2_ synthesized film was previously reported and a rough but fairly uniform grain size distribution was observed [42].

Reported BET analysis of titania NTs has suggested that the surface area is between 12 [12] and 38 m^2^ g^−1^ [49]. Given that the NTs by mass are much less than 1.0 mg cm^−2^, it is not then possible to measure the specific surface area on these samples, but it will be less than 0.038 m^2^. The P25 TiO_2_ has a specific surface area of 50 m^2^ g^−1^ as the free powder. Necking of the particles occurs through partial sintering at 450 °C, so the surface area will be somewhat less that of the free particles; however, the loading is 1.0 mg cm^−2^ of P25 (<0.05 m^2^). The N,F-TiO_2_ as free powder has a surface area of 136 m^2^ g^−1^, but the film thickness is around 1.5 µm, with 160 μg cm^−2^ (<0.02 m^2^).

**Figure 2:**
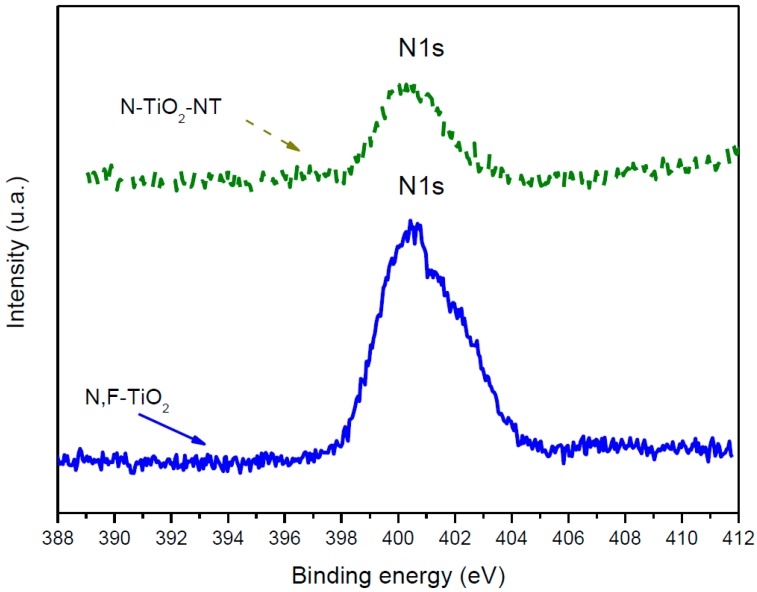
XPS spectra of the N 1s core level obtained for N,F-TiO_2_ and N-TiO_2_-NT.

XPS analysis was used to confirm nitrogen incorporation in the nanotube electrodes (Figure 2).

Analysis of the N 1s region from the XPS spectra (Figure 2) shows a wide peak centered with binding energy at 399.5 eV indicating that nitrogen is incorporated at interstitial sites [42]. The presence of fluoride in the N,F-TiO_2_, using this preparation method, was previously confirmed by a peak centered at 688 eV [42]; however, there was no significant peak in the analysis of the materials fabricated in this work. The concentration of nitrogen in the N,F-TiO_2_ was determined to be 1.5 atom%. The concentration of nitrogen in the N-TiO_2_-NT was 0.5 atom%. Asahi et al. reported an optimal N concentration of 0.25 atom% with samples prepared by sputter deposition [23]. Delegan et al. reported an optimal N doping at 0.4 atom% for band gap narrowing with no further effect at higher loadings [50]. Therefore, the N doping level of 0.5 atom% in the N-TiO_2_-NTs is similar to that reported for optimal doping. According to the literature, peaks in the range of 400–402 eV may be due to molecularly chemisorbed N_2_ [38,45] whilst NO_2_ and NO_3_ have been reported to occur at higher energies 403–408 eV [16,51,52]. Other groups have observed the N 1s peak alone at 399–400 eV and assigned it as NO [38]. However, the absence of peaks beyond 407 eV indicates the absence of chemisorbed NO or NO_2_ species [53]. In general, the N 1s peak at ca. 400 eV is typically assigned to the interstitial nitrogen dopant while the peak at ca. 396 eV is associated to the substitutional nitrogen dopant. However, there is no consensus about which kind of doping is more efficient for giving rise to visible light activity [34].

**Figure 3:**
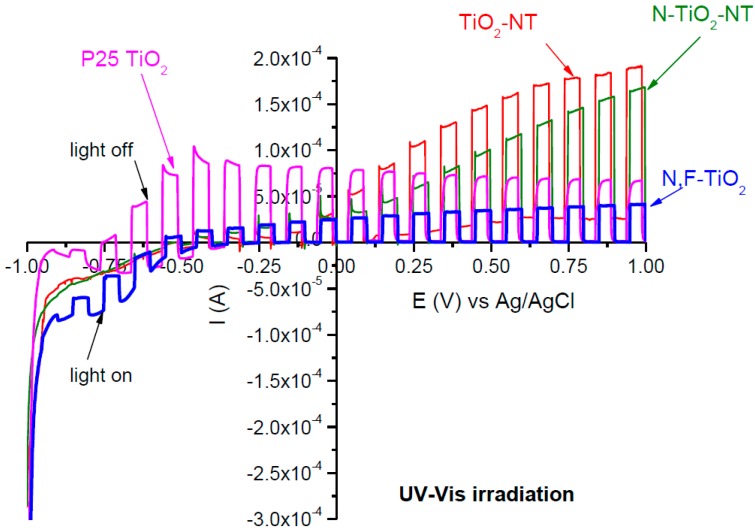
Linear sweep voltammograms for nanotube (TiO_2_-NT and N-TiO_2_-NT) and nanoparticle (P25 and N,F-TiO_2_) titania electrodes in ¼ strength solution under chopped (10 s light on/ off) UV-Vis irradiation. The potential was swept from negative to positive with a sweep rate of 5 mV s^−1^.

To study the photoelectrochemical properties, linear sweep voltammetry (LSV) was carried out under chopped UV-Vis irradiation. The supporting electrolyte was a ¼ strength Ringers solution. The current-potential behavior of the different materials is shown in Figure 3.

All samples showed negligible dark anodic current under UV-Vis irradiation, which is typical of n-type semiconductor electrodes in contact with an electrolyte. It has widely been reported that electron transport, in particulate electrodes, occurs by diffusion since the small size of the particles does not allow the formation of the depletion layer across the particle [54,55,56]. Moreover, as a consequence of inter-particle boundaries due to their porous structure, many localized states may exist leading to charge carrier recombination. Therefore, the photocurrent is not expected to be dependent on the applied potential. However, the Fermi level of the supporting electrode is potential dependent [57,58] and the application of a positive potential leads to a decrease in the Fermi level of the supporting electrode and consequently, to the increase in the efficiency of electron drift from the irradiated nanoparticulate film [15].

It must be noted that both nanotube electrodes (undoped and N-doped) show the highest values of photocurrent under UV-Vis illumination in comparison with both nanoparticle electrodes (P25 and N-doped sol-gel) at the same anodic potential. This is in agreement with other workers [11,12,15] who reported a higher photocurrent of titania nanotubes as compared to P25; and others who observed a higher photocurrent of titania nanotubes compared to titania sol-gels or films [46,59,60,61]. However, it should be noted that the onset potential for anodic photocurrent is more positive with the NT electrodes as compared to the P25 or N,F-TiO_2_ electrodes.

Several authors have pointed out that despite the nanotubular morphology, these nanostructured materials give a lower surface area compared to that of mesoporous nanoparticle thick films (e.g., P25), yet the NT electrodes give a higher photocurrent response [11,12,15]. In nanoparticulate films the particles are partially sintered and electron diffusion to the supporting electrode follows a convoluted pathway via particle to particle across grain boundaries. Also, the electrolyte penetrates into the mesoporous film and conduction band electrons may be lost to surface recombination reactions resulting in relatively low photocurrent, as compared to compact oxide electrodes. The aligned nanotube morphology provides a more direct pathway for the electrons to transfer to the supporting electrode through the continuous TiO_2_ tube wall. Also, the NTs are grown from the titanium substrate which favors good electron transfer from the NTs to the metal support [59]. It is also worth noting that there was only a small difference in photocurrent response for the doped and undoped nanotube electrodes.

**Figure 4:**
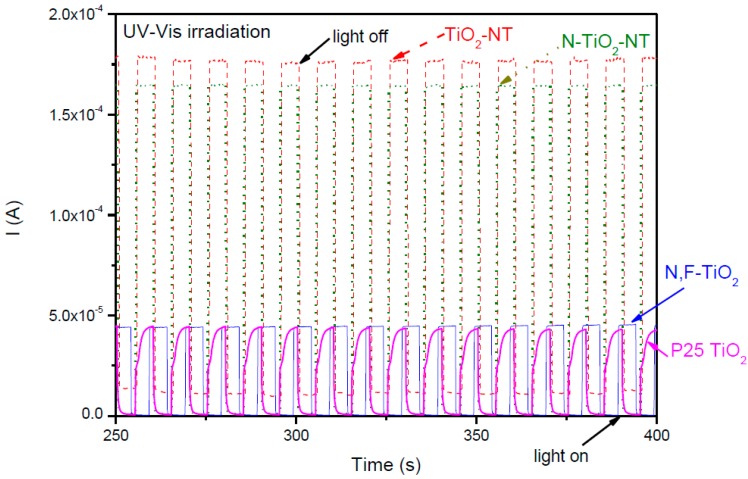
Current-time behavior of nanotube (TiO_2_-NT and N-TiO_2_-NT) and nanoparticle (P25 and N,F-TiO_2_) electrodes in ¼ strength solution under chopped UV-Vis irradiation. UV-Vis exposure time: 5 s. Potential bias: +1.0 V. 3rd cycle of current measurement for each electrode.

The current-time behavior of all the electrodes at a fixed potential of +1.0 V is given in Figure 4. The same trends in photocurrent intensity were observed at fixed potential as were observed in the LSV. Both the P25 and N,F-TiO_2_ samples gave a similar photocurrent response. The NT samples gave the highest photocurrent values. The third cycle of amperometry is represented for each electrode. No decrease in photocurrent is observed between the first (data not shown) and the third cycle in any sample, therefore there is no observed decrease in the photocurrent activity in repeat runs.

**Figure 5:**
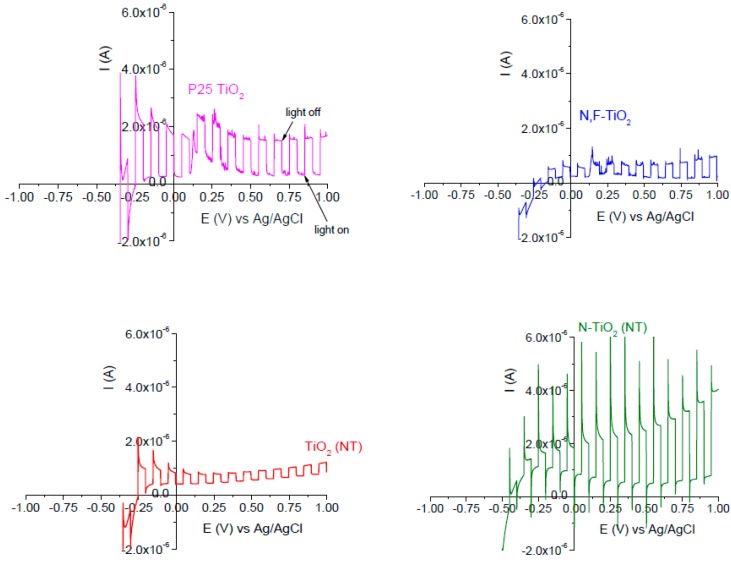
Linear sweep voltammograms for nanoparticle (P25 and N,F-TiO_2_) and nanotube (TiO_2_-NT and N-TiO_2_-NT) electrodes in ¼ strength solution under chopped visible irradiation (10 s light on/off). The potential was swept positive at a sweep rate of 5 mV s^−1^.

Linear sweep voltammetry was also carried out under visible only irradiation (Figure 5). The photocurrent response under only visible light is two orders of magnitude lower than that observed under UV-Vis irradiation (Figure 4) which agrees with previous results [62]. This would indicate that the visible light activity for water oxidation is two orders of magnitude lower than that under band gap excitation. Previously, it has been reported that the hydroxyl radical is the main species responsible for *E. coli* inactivation with UV excited nonmetal doped TiO_2_ [63]. If the anodic photocurrent is a measure of water oxidation, and formation of the hydroxyl radical, therefore, one might expect a rate of two orders of magnitude lower under visible as compared to UV (not considering direct photolytic inactivation) and as such, no inactivation would be observed under visible irradiation in the timescale of the experiments. The nitrogen doped titania nanotube sample (N-TiO_2_-NT) showed the highest photocurrent (*I*_ph_) under visible irradiation. The N,F-TiO_2_ electrode also showed a small visible photocurrent response. Interestingly, the P25 shows a small visible photocurrent response but it is noted that P25 is an 80:20 mixture of anatase and rutile, with the rutile band gap at 3.0 eV, just into the visible region (413 nm).

The N-TiO_2_-NT photoresponse is also higher than that observed for the other doped titania sol gel sample (N,F-TiO_2_). Again, it suggests that nanotubular structure offers a less resistant path for the electrons to reach the supporting electrode, and consequently, it leads to a more effective separation of the electron—hole pairs. In addition, if both undoped and doped nanoparticulate samples are compared, their photocurrent response is similar.

It must be highlighted that the photocurrent response under visible irradiation is only a fraction of the band gap response observed under UV-Vis irradiation for the N-TiO_2_-NT electrode (and all electrodes). This correlates with previous work investigating the photoelectrochemical response of N,F-TiO_2_ [62] where it was found that the visible light photocurrent was only a fraction of the band gap response and the photocurrent action spectrum did not correlate to the optical absorbance spectrum for the material; however, the open circuit photopotential gave a better correlation to the optical spectra.

**Figure 6:**
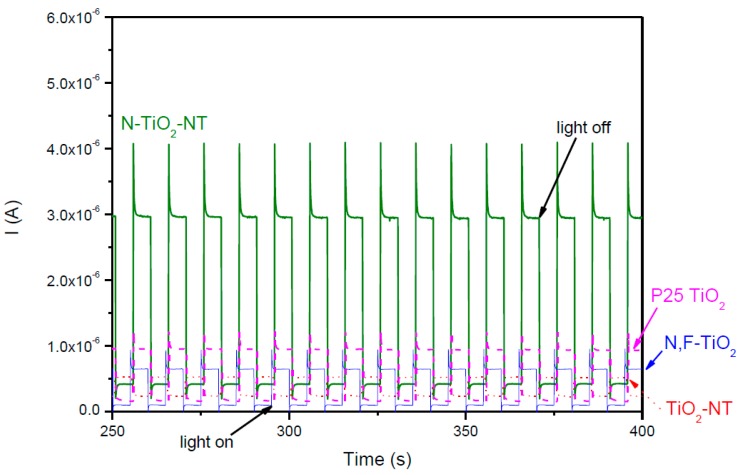
Current-time behavior of nanotube (TiO_2_-NT and N-TiO_2_-NT) and nanoparticle (P25 and N,F-TiO_2_) electrodes in ¼ strength solution under chopped visible irradiation (light on/off 10 s) at fixed potential (+1.0 V). Data shown is from the third cycle of current measurement for each electrode.

The current-time behavior of all the electrodes, shown in Figure 6, is typical of an n-type semiconductor under chopped illumination. An initial anodic photocurrent spike (*I*_ph in_) is instantly observed under exposure to illumination which corresponds to separation of photogenerated e^−^-h^+^ pairs. Then, photocurrent decay is observed until a steady-state photocurrent (*I*_ph st_) is reached, due to surface charge carrier recombination and/or accumulation of holes at the surface. A cathodic photocurrent spike (*i*^−^_in_) is observed when the light is turned off, representing back reaction of conduction band electrons with the hole trap sites at the surface [33,64]. In this case, a higher recombination might be suggested by the observed decay in the initial *I*_ph_ produced in the instant of illumination for N-TiO_2_-NT under visible irradiation as compared to UV-Vis irradiation (Figure 4). 

### 2.2. Electrochemically Assisted Photocatalytic Disinfection

**Figure 7:**
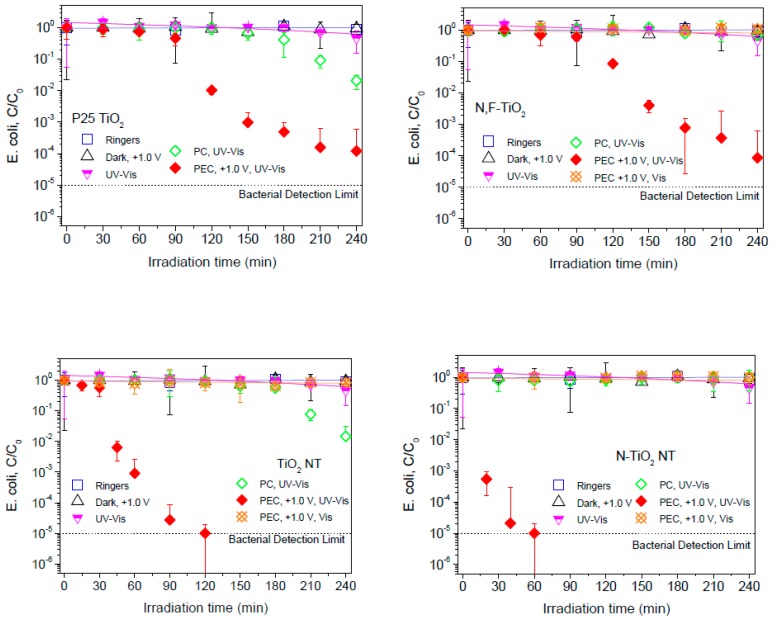
Photocatalytic and electrochemically assisted photocatalytic inactivation of *E. coli* for the different electrodes in ¼ strength solution under UV-Vis irradiation, and visible only irradiation for N,F-TiO_2_, TiO_2_-NT, and N-TiO_2_-NT electrodes. The applied potential was +1.0 V for electrochemically assisted photocatalysis. PC = Photocatalysis, Ringers = dark control, Dark, +1.0 V = electrochemical control, UV-Vis = light control. All the experiments have been performed in triplicate using the same electrode.

Figure 7 shows *E. coli* inactivation obtained with the different photoelectrodes: P25 TiO_2_, N,F-TiO_2_, TiO_2_-NT, and N-TiO_2_-NT. Firstly, different control experiments were carried out. The chemical composition of the supporting electrolyte, electrochemical oxidation under an electric potential bias of +1.0 V, and photolysis did not lead to any significant decrease in the viable concentration of *E. coli* after 4 hours of treatment for any of the electrodes analyzed.

The extent of bacterial inactivation for photocatalysis (electrodes at open circuit) was small over the timescale of the experiments. This is to be expected as the irradiated surface area to volume ratio (Incident Illuminated Density, ICD) for this reactor system is 0.066 cm^2^ cm^−3^. In a previous study, complete inactivation was observed at around 100 min for *E. coli* with photocatalysis on P25 TiO_2_ films in a stirred tank reactor with an ICD of 0.28 cm^2^ cm^−3^ [65]. However, the photocatalytic bacterial inactivation is notably improved by the application of a positive electrical potential for all the TiO_2_ electrodes under UV-Vis irradiation. Dunlop et al. [6] reported that the rate of inactivation of *E. coli* was increased using an applied potential of +1.0 V (SCE) as compared to photocatalysis alone for particulate electrodes (Degussa and Aldrich (anatase)) as well as an increase in bacterial inactivation with application of a positive bias. Also, Dunlop et al. [66] reported that the application of an external electrical bias significantly increased the rate of photocatalytic disinfection of *C. perfringens* spores on particulate electrodes (P25). Thus, it suggests that the increase in inactivation efficiency for nanoparticulate electrodes is probably due an improvement of mass transport of the negatively charged bacteria to the positively charged photoanode by electromigration [8,9,10].

Comparing the photocurrent at fixed potential (+1.0 V) under UV-Vis irradiation (Figure 4) it is clear that the NT electrodes give a much better response i.e., 175 μA for the TiO_2_-NT and 165 μA for the N-TiO_2_-NT electrodes, as compared to less than 50 μA for the NF-TiO_2_ and P25 TiO_2_ electrodes. Comparing the photocurrent at fixed potential (+1.0 V) under visible only irradiation (Figure 6), the N-TiO_2_-NT electrode gives the highest photocurrent (3 μA) as compared to P25 TiO_2_ (1 μA), N,F-TiO_2_ (0.6 μA) and TiO_2_-NT (0.5 μA). The order of photocurrent magnitude correlates with the observed electrochemically assisted photocatalytic disinfection rates under UV-Vis irradiation (Figure 7) in that both NT electrodes reach a 5 log inactivation within the timescale of the experiments (120 min for the TiO_2_-NT and 60 min for the N-TiO_2_-NT), but neither the N,F-TiO_2_, nor the P25 TiO_2_ electrodes achieve a 5 log kill within 240 min. None of the electrodes gave any significant inactivation of bacteria under only visible irradiation. It is noted that the visible light photocurrent is two orders of magnitude lower than the photocurrent observed under UV-Vis irradiation. The correlation with photocurrent and disinfection rate fails when comparing the N-TiO_2_-NT and the TiO_2_-NT under UV-Vis irradiation. The time taken to reach a 5 log inactivation for the N-TiO_2_-NT is only 30 min while it takes 120 min for the TiO_2_-NT under UV-Vis irradiation. Both NT electrodes give similar UV-Vis photocurrent at +1.0 V, but the N-TiO_2_-NT electrode gives a greater visible only photocurrent, yet this is only 2% of the UV-Vis photocurrent. If one considers that the main species responsible for photocatalytic inactivation of bacteria under UV irradiation on TiO_2_ is the hydroxyl radical [63], then one would expect the time taken for inactivation on both NT electrodes to be similar, based on photocurrent. It is not clear why the N-TiO_2_-NT electrode should give a much faster rate of inactivation, but this must be related to the mechanism of disinfection. Differences in the attachment/association of bacteria to the surface of the catalyst should not be excluded as the materials may have different properties concerning surface interactions. Additionally, Hamilton et al. [62] previously reported that the mid-gap state introduced by N-doping of titania could yield some visible photocatalytic activity by the visible light excitation of electrons from the mid-gap state to the conduction band leading to the reduction of molecular oxygen to superoxide and hydrogen peroxide, and/or the oxidation of superoxide by visible light generated holes in the mid-gap state to yield singlet oxygen. The additional ROS generated under visible excitation, in combination with the hydroxyl radicals generated by UV excitation, yields a much faster inactivation rate as compared to the UV photocatalytic mechanism alone. Previous work reported on the activity of N-TiO_2_ thin-films produced using atmospheric-pressure chemical vapor deposition (APCVD) with tert-butylamine as the nitrogen source [67]. They attributed the enhancement of the UV activity of the N-TiO_2_ to surface N species which, were not stable, leading to a decrease in activity over time. However, in this work the disinfection experiments were undertaken in triplicate with no decrease in disinfection efficiency observed between the experiments. Furthermore, no decrease in the photocurrent response was observed in repeat measurements, suggesting that these materials are relatively stable.

Further research is required to elucidate the difference in the electrochemically assisted photocatalytic disinfection mechanism on N-TiO_2_-NT and TiO_2_-NT. Nevertheless, the N-TiO_2_-NT electrode gives a much faster rate of disinfection under UV-Vis irradiation than any of the other materials studied.

## 3. Experimental Section

### 3.1. TiO_2_ Electrode Preparation

Ti foil (Sigma-Aldrich, Irvine, UK, 0.127 mm, 99.7%) was cut into 2.5 × 2.0 cm^2^ pieces and cleaned in an ultrasonic bath in methanol for 15 min. An area of ca. 1 cm^2^ was exposed for coating or iodization. 

Two particulate photoelectrodes were prepared by: (i) electrophoretic coating of a TiO_2_ suspension. A Ti foil was submerged in a 1% P25 TiO_2_:CH_3_OH suspension of P25 TiO_2_ (P25 Evonik industries, Rhine-Main Germany, CH_3_OH, 99.9% Sigma Aldrich, Scotland, UK) and an electric potential bias of +20 V vs. a Pt paddle (Windsor scientific, Berkshire, UK) used as the anode was applied for 15 s [15]. The samples were annealed at 450 °C for 1 h in air with a heating rate of 2 °C min^−1^; (ii) dip-coating of N and F co-doped TiO_2_ suspensions (N,F-TiO_2_) onto a Ti foil. This catalyst sol was prepared according to Pelaez et al. [42], using titanium (IV) isopropoxide (TTIP, 97%, Aldrich, Irvine, UK) as the titania precursor and anhydrous ethylenediamine (EDA, Fisher, Loughborough, UK) as the nitrogen source. The chosen concentration of the non-ionic fluorosurfactant in molar ratio corresponded to five. The sol was deposited onto the Ti substrate at a withdrawal speed rate of 4.9 mm s^−1^. The samples were annealed at 400 °C for 30 min in air. The temperature was increased at a ramp rate of 1 °C min^−1^ and cooled down at 4 °C min^−1^. 

Titania nanotubes were grown by the electrochemical oxidation of Ti in a fluoride-based electrolyte according to Dale et al. [15]. Ti foil pieces were anodized in a custom designed two electrode electrochemical cell made of *Perspex* with a 100 mL capacity. Each Ti foil piece was fixed in the cell with copper back-plate electrical contact. An O-ring was used to seal the foil in the cell, leaving an area of ca. 1 cm^2^ exposed to the electrolyte (1 M Na_2_SO_4_ + 0.12 M NaF in aqueous solution). A platinum electrode served as the counter electrode. The distance between the two electrodes was 3 cm. Conducting wires soldered to the copper back-plates allowed connection to a power supply. The Ti foil was treated at constant potential of +25 V for 4 h using the power supply. After anodization, the Ti foil was ultrasonicated in methanol for 5 min to wash off any remnant salts from electrolyte. Undoped titania samples were annealed in air at 450 °C at the ramp rate of 2 C min^−1^ for 1 h (TiO_2_-NT) and those doped with nitrogen (N-TiO_2_-NT) were annealed at 450 °C at the same ramp rate but in NH_3_ (BOC gas and gear, BOC, Belfast, UK). All the samples were made into electrodes by cleaning part of the Ti foil to attach a copper wire using silver loaded epoxy. Afterwards, the contact, wire and titanium were masked with negative photoresist (KPR, Cassio chemicals, Hertfordshire, UK) which was cured under UV-A exposure for 10 min. The electrodes were finally sealed with epoxy resin (Araldite) leaving an area of TiO_2_ of 1 cm^2^ exposed.

### 3.2. Materials Characterisation

Scanning electron microscopy (SEM, FEI Quanta 200, FEI Eindhoven, The Netherlands) was used to confirm the formation of nanotubes for the TiO_2_-NT and N-TiO_2_-NT electrodes. Chemical composition analysis was undertaken by X-ray photoelectron spectroscopy (XPS) with a Kratos Axis Ultra employing an Al Kα source (Kratos Analytical, Eppstein, Germany). The binding energies of the samples were calibrated relative to the C 1 s peak at 284 eV. 

### 3.3. Photoelectrochemical Analysis

**Figure 8:**
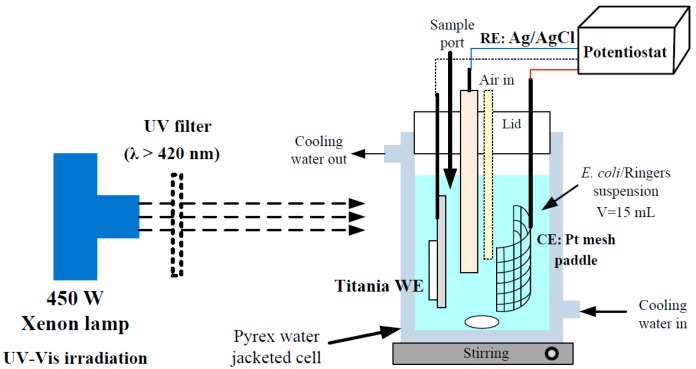
Photoelectrochemical cell.

Linear sweep voltammetry (LSV) was used to determine the current-potential characteristics of the electrodes under chopped irradiation. Photocurrent measurements were recorded at a scan rate of 5 mV∙s^−1^ sweeping from −1.0 to +1.0 V under irradiation. Photocurrent-time measurements at a fixed potential (+1.0 V) were also carried out. A shutter (Unblitz, WMM-T1, Vincent associates, Rochester, NY, USA) was also used to chop the light. The titania samples were used as the working electrodes (WE), a Pt mesh paddle (5.9 cm^2^) was used as the counter electrode (CE) and an Ag/AgCl electrode was used as the reference electrode (RE) (Figure 8). An electrochemical workstation with PC control (Autolab PGStat 30, Metrohm UK, Runcorn, UK) provided potentiostatic control. Quarter strength Ringers solution (Oxoid, Fisher, Hampshire, UK) was used as electrolyte, consisting of 2.25 × 10^3^ mg dm^−3^ NaCl, 1.05 × 10^2^ mg dm^−3^ KCl, 1.2 × 10^2^ mg dm^−3^ CaCl_2_, and 50 mg dm^−3^ NaHCO_3_ in deionized water [6]. 

### 3.4. Photoreactor Configuration

The photoreactor used was a 35 cm^3^ Pyrex water-jacketed reactor (Figure 8). The working volume used corresponded to 15 mL. Air was bubbled in through a Pasteur pipette suspended in the working solution. Distilled water was pumped from a thermostated water tank and circulated round the reactor water jacket. The suspension was magnetically stirred to ensure effective mixing and the temperature was maintained at 25 °C throughout the experiments. The suspension was irradiated with UV-Vis using a 450 W xenon lamp (Horiba Jobin Yvon, Horiba UK LTD, Northampton, UK) full spectrum as solar simulated irradiation placed 4 cm away from the reactor. The incident light intensity reaching the suspension was determined to be 197 W m^−2^ (measured between 200–800 nm using a spectral radiometer, Gemini 180, Horiba Jobin Yvon, Horiba UK LTD, Northampton, UK). For visible light only, a UV filter (λ > 420 nm) (UQG Optics Ltd., Cambridge, UK) was placed between the reactor and the irradiation source. The incident light intensity corresponded to 150 W m^−2^.

The same reactor was used for the electrochemically assisted photocatalytic experiments with the photoanode under a fixed potential of +1.0 V vs. Ag/AgCl. The photocurrent was recorded against time for each experiment. Quarter strength Ringers solution was used as the working suspension. 

### 3.5. Bacterial Growth and Detection 

*Escherichia coli* K12 was used as the model microorganism for the inactivation experiments. Fresh liquid cultures were prepared by inoculation in a Luria-Bertani nutrient medium (Sigma-Aldrich, Irvine, UK) and incubation at 37 °C for 24 h under constant stirring on a rotary shaker. The cell density of the culture was checked prior to the experiment by measuring its absorbance at 520 nm as prediction of the initial bacterial concentration. Cells were harvested by centrifugation (at 4000 rpm for 10 min) washing twice the bacteria with sterile ¼ strength Ringers solution before resuspension in the same solution. Finally, a volume of the bacterial suspension corresponding to 0.15 mL was diluted in 15 mL of sterile ¼ strength Ringers solution to start the experiments at an initial concentration approximately 10^6^ CFU mL^−1^. The bacterial inactivation was followed by evaluating the concentration of viable bacteria in the samples taken throughout the reaction. The quantification was carried out following a standard serial dilution procedure. Serial dilutions in sterile ¼ strength Ringers solution were carried out prior to spotting 10 μL drops of each decimal dilution 4 times on LB nutrient agar plates. At longer irradiation times (low bacterial concentrations), 2 drops of 100 μL were obtained from the working suspension of the undiluted suspension were plated directly onto LB agar to reduce the limit of bacterial detection to 10 CFU mL^−1^. All LB agar plates were incubated at 37 °C for 24 h, and the number of colonies were manually counted. Key experiments were also repeated three times to test the reproducibility of the disinfection results. Full details of components of the LB medium are available elsewhere [6].

## 4. Conclusions

Aligned self-organized titania nanotubes were grown on titanium foil by anodic oxidation in fluoride containing electrolyte (TiO_2_-NT). The nanotube samples were doped with nitrogen by annealing in ammonia. Nanoparticulate films of N,F-TiO_2_ and P25 were prepared for comparison purposes. 

This study demonstrates that electrochemically assisted photocatalysis is much more effective than photocatalysis alone (electrodes at open circuit) for the inactivation of *E. coli* under solar simulated UV-Vis irradiation for all of the different electrodes tested. No significant inactivation of bacteria was observed under only visible light irradiation of any of the materials tested, with or without applied potential. The photocurrent response of the electrodes at fixed potential under UV-Vis irradiation correlated well with the disinfection rates observed under the same conditions, in that the NT electrodes gave a much higher photocurrent response than the nanoparticulate electrodes and a much better disinfection efficiency. The most interesting finding of this work was that the N-TiO_2_-NT electrode gave a much better disinfection rate at +1.0 V under UV-Vis irradiation as compared to any of the other materials studied. The time taken for a 5 log kill on the N-TiO_2_-NT was half that observed for the TiO_2_-NT electrode. However, the UV-Vis photocurrent was slightly higher for the TiO_2_-NT electrode at a fixed potential. This suggests that the N doping generates mid gap states which yield visible light activity through the formation of superoxide and singlet oxygen. Although, these ROS are less effective for the inactivation of bacteria than hydroxyl radical (produced under UV excitation), their presence should not be dismissed. Also, the visible light excitation of mid gap excitation does not generate significant photocurrent (only 2% of the UV-Vis response). The higher UV-Vis disinfection efficiency of the N-TiO_2_-NT electrodes must be due to differences in the disinfection mechanism and further research is required to elucidate these differences. Nevertheless, N doping of titania nanotubes gives electrodes with excellent UV-Vis activity for the electrochemically assisted disinfection of water.
